# Structural Analysis and Immuno-Stimulating Activity of an Acidic Polysaccharide from the Stems of *Dendrobium nobile* Lindl.

**DOI:** 10.3390/molecules22040611

**Published:** 2017-04-10

**Authors:** Jun-Hui Wang, Shu-Rong Zuo, Jian-Ping Luo

**Affiliations:** School of Food Science and Engineering, Hefei University of Technology, Hefei 230009, China; zuoshurong727@163.com

**Keywords:** *Dendrobium nobile* Lindl., polysaccharide, acidic polysaccharide, immunological activities

## Abstract

*Dendrobium nobile* Lindl., an epiphytic herb distributed in the Southeast Asia, is used as a tonic and antipyretic herbal medicine in China. In this study, a water-soluble acidic heteropolysaccharide, DNP-W4, containing mannose, glucose, galactose, xylose, rhamnose, and galacturonic acid, in the molar ratios of 1.0:4.9:2.5:0.5:1.0:0.9, was obtained from the stems of *Dendrobium nobile* Lindl. Using methylation analysis, partial acid hydrolysis, pectolyase treatment, NMR, and ESI-MS, the structure of DNP-W4 was elucidated. The obtained data indicated that DNP-W4 was a complex heteropolysaccharide and possessed a backbone composed of (1→4)-linked β-d-Glc*p*, (1→6)-linked β-d-Glc*p*, and (1→6)-linked β-d-Gal*p*, with substitutes at *O*-4/6 of Glc*p* residues and *O*-3 of Gal*p*. The branches of DNP-W4 were composed of terminal Man*p*, (1→6)-linked β-d-Man*p*, (1→3)-linked β-d-Glc*p*, β-d-Glc*p*, β-d-Gal*p*, (1→4)-linked α-d-GalA*p*, (1→2)-linked α-L-Rha*p*, and Xyl*p*. DNP-W4 had little immunological activities, but its derivatives had immuno-stimulating activities to some extent.

## 1. Introduction

In the recent years, polysaccharides from medicinal plants have attracted great attention in the food industry owing to their wide variety of bioactivities, especially antioxidant and immuno-stimulating activities, indicating that polysaccharides from medicinal plants can offer a safe and natural source of antioxidants and immune-stimulants for application in the food industry [1,2,3]. They may be added to foods as additives or consumed directly as functional foods. The genus *Dendrobium* (Orchidaceae), which includes more than 1600 species, is a group of edible and medicinal plants in the Southeast Asia [4]. It is reported that there are 74 species and two varieties of *Dendrobium* plants in China [5]. The dried and the fresh stems of several *Dendrobium* species are used in traditional medicine or as materials to make herbal teas for thousands of years in China. It is claimed that the compounds from *Dendrobium* plants might enhance the immunity, nourish the stomach, reduce fever, relieve throat inflammation, and prevent the development of cataracts [4,5,6]. Recently, different pharmacological experiments have been carried out with polysaccharides from *Dendrobium* plants, which have been demonstrated to have bioactive components and showed to possess immuno-stimulatory, anti-tumor, antioxidant, and anti-mutagenic activities [7,8,9,10]. Moreover, to verify the activity of *Dendrobium* plants, some water-soluble polysaccharides from *Dendrobium* species, including *D. candidum* [11], *D. aphyllum* [12], *D. officinale* [13], *D. huoshanense* [8,10], *D. denneanum* [14] and *D. nobile* [15,16,17], have been isolated and purified, and their structures were identified.

*Dendrobium nobile* Lindl. (Chinese name “Jin-Chai-Shi-Hu”) is one of the most popular *Dendrobium* plants, widely distributed in the southwest of China, that has been recorded in the Chinese Pharmacopoeia [6,18] as one of the original materials of “Shi Hu.” Crude polysaccharides extracted from *D. nobile* have been shown to trigger anti-tumor, antioxidant and anti-cataract activities, indicating their potential as antioxidants and immune-stimulants to be used in the food industry [9,19]. To elucidate the pharmacological qualities of *D. nobile*, much research has been carried out on the low molecular compounds, including alkaloids, bibenzyls, stilbenoids, glycosides, sesquiterpenes, fluorenones, and phenanthrenes [20,21,22,23,24,25]. Recently, the polysaccharides from *D. nobile* proved to be some of the main active components that possess immuno-stimulating, anti-tumor, antioxidant and anti-mutagenic activities [9,26]. In order to better understand the use of *D. nobile*, a systematic study on the polysaccharides from *D. nobile* was performed, including extraction, isolation, purification, and identification of structure and bioactivities [9,15,16,17]. In this paper, the structure characterization and immunological activities of a new acidic polysaccharide from the stems of *D. nobile* were evaluated and reported.

## 2. Results and Discussion 

### 2.1. Isolation, Purification and Composition Analysis of DNP-4W

**Figure 1:**
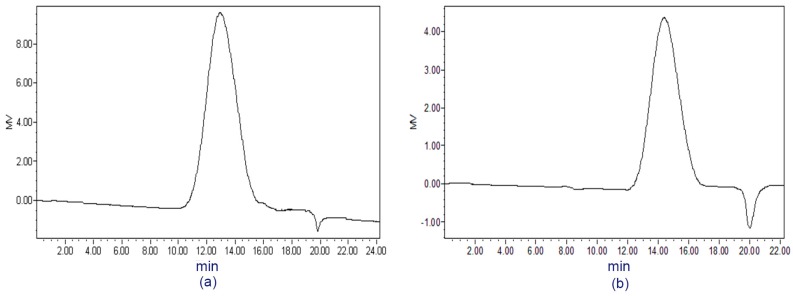
HPGPC chromatogram of DNP-W4 (**a**) and DNP-W4E (**b**) from *D. nobile*.

The water extract from the dried stems of *D. nobile* was precipitated with 4 vols. EtOH to obtain the crude polysaccharide DNP-W. After successive separations by DEAE-cellulose anion exchange and Sephacryl S-200 gel filtration chromatography, the carbohydrate fraction DNP-W4 was successfully separated. The result of the high performance gel-permeation chromatography (HPGPC) showed that DNP-W4 had only one symmetrical peak (Figure 1a). The molecular mass of DNP-W4 was estimated to be 5.0 × 10^5^ Da in reference to the standard dextrans, and showed a specific rotation of ${\left[\alpha \right]}_{D}^{20}$ −10.1 (*c* 0.5, H_2_O). No absorption was noted at 280 nm; a negative response to the Lowry method [27] confirmed that DNP-W4 did not contain protein.

The *m*-hydroxybiphenyl method [28] determined that DNP-W4 contained 8.6% uronic acid. After reduction with CMC–NaBH_4_, the carboxyl-reduced polysaccharide DNP-W4R was obtained. A combination of sugar composition analysis of DNP-W4 and DNP-W4R revealed that DNP-W4 contained mannose, glucose, galactose, xylose, rhamnose, and galacturonic acid in the molar ratios of 1.0:4.9:2.5:0.5:1.0:0.9 (Table 1). The ratio of GalA was established by the increase of Gal content in DNP-W4R. All the glycosyl residues were D configuration, except rhamnose which was L configuration [29].

### 2.2. Structural Characterization of DNP-W4

**Figure 2:**
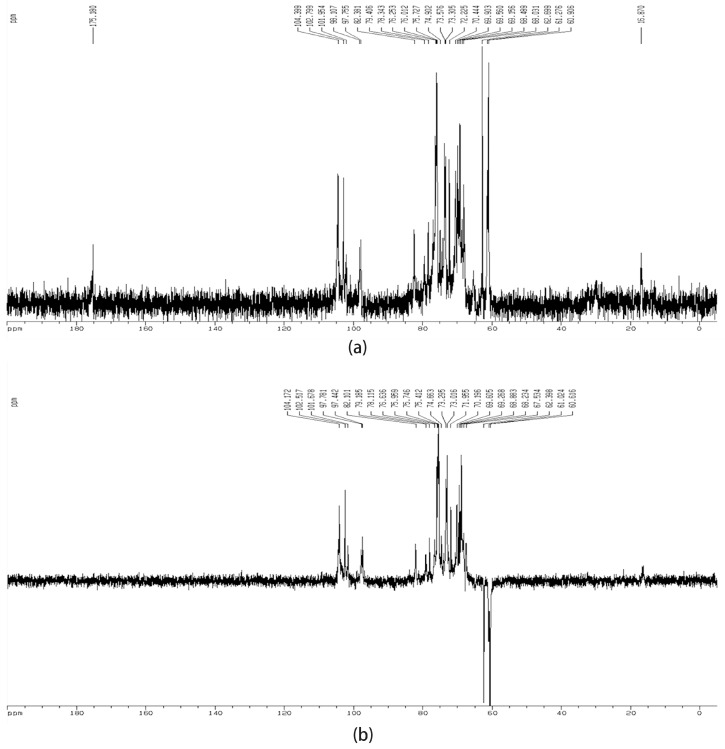
^13^C-NMR spectra of DNP-W4 (**a**) and DEPT (**b**) from *D. nobile*.

The anomeric configuration of each residue in DNP-W4 was elucidated with the ^13^C-NMR spectra, based on the component analysis, linkage analysis and on literature values [13,30,31,32]. The ^13^C-NMR spectra of DNP-W4 (Figure 2a) showed a signal of *C*-methyl at δ 16.8. The signal at δ 104.3 in the anomeric carbon region, could be attributed to C-1 of β-Glc*p*, δ 102.8 to C-1 of β-d-Gal*p*, δ 101.9 to C-1 of β-d-linked Man*p*, δ 98.1 to C-1 of α-(1→4)-linked GalA*p*, δ 97.7 to C-1 of α-(1→4)-linked Rha*p*, and δ 96.8 to C-1 of α-d-linked Xyl*p*, respectively.

Both DNP-W4 and its carboxyl-reduced derivatives DNP-W4R were methylated. The methylation analysis of DNP-W4 and DNP-W4R showed that DNP-W4 contained 1,4-linked GalA and Rha residues together with other neutral residues (Table 2). DNP-W4 was treated with pectolyase to remove the structures of pectin, and the resulting product DNP-W4E was analyzed by TLC and GC. The results (Table 1) showed that most of Rha, Xyl and GalA residues were removed by pectolyase, and in HPGPC (Figure 1b), the molecular mass of DNP-W4E showed to be lower (4.1 × 10^5^ Da). It is probable that the pectolyase-treatment caused a substantial loss of molecular mass. These results indicate that the pectic component of Rha and GalA might be a part of DNP-W4. When observing the effects of the absence removal of Xyl in DNP-W4E, it should be considered that the Xyl residues in DNP-W4 should form short chains and be tightly attached to the pectin chain of RG (rhamnogalacturonan) core. The loss of Xyl could be explained by the cleavage of the pectin chain of the RG core by pectolyase-treatment and the consequent loss during dialysis (molecular mass of xylosyl chains <3500).

Mild acid hydrolysis of DNP-W4 gave a pure fragment HDNP-W4 with a molecular weight of 3.8 × 10^5^. HDNP-W4 showed to be composed of mannose, glucose, galactose, xylose, rhamnose, and galacturonic acid in the molar ratios of 1.8:14.1:4.9:1.1:1.0:0.8, respectively (Table 1). The results of the linkage analysis of HDNP-W4 (Table 2) indicated that, compared with the methylation analysis of DNP-W4, the content of terminal Glc*p* residues, 1,3-linked Glc*p* residues, terminal Man*p* residues, 1, 6-linked Man*p* residues, terminal Xly*p* residues, 1,2-linked Rha*p*, and 1,4-linked Gal*p*A residues had decreased, and that the molar ratios of 1,6-linked Glc*p*, 1,6-linked Gal*p* and 1,4-linked Glc*p* residues had increased. These results suggested that Xly*p* residues, Man*p* residues, and some of Glc*p* residues might be present at side chains, attached to the increased glycosyl residues. Further methylation analysis showed that DP1 (oligomer product in hydrolysis) contained a high ratio of 1,2-linked Rha*p* and 1,4-linked Gal*p*A (Table 2), indicating that the RG region had a large proportion of side chains that might form a tight hairy region.

DNP-W4 was oxidized with 0.015 M sodium metaperiodate (NaIO_4_) at room temperature in the dark for 7 days. A total of 1.25 mol NaIO_4_ was consumed per mole of sugar residues, based on the average molar mass (180 Da) of a glycosyl residue. The production of formic acid was 0.47 mol. It was thus deduced that the nonreducing terminal residues or (1→6)-linked glycosyl bonds accounted with 47%, with (1→2)-/(1→4)-linked and (1→3)-linked glycosyl bonds accounting with 22% and 31%, respectively. These values were in approximate agreement with the theoretical values calculated on the basis of the methylation data.

**Figure 3:**
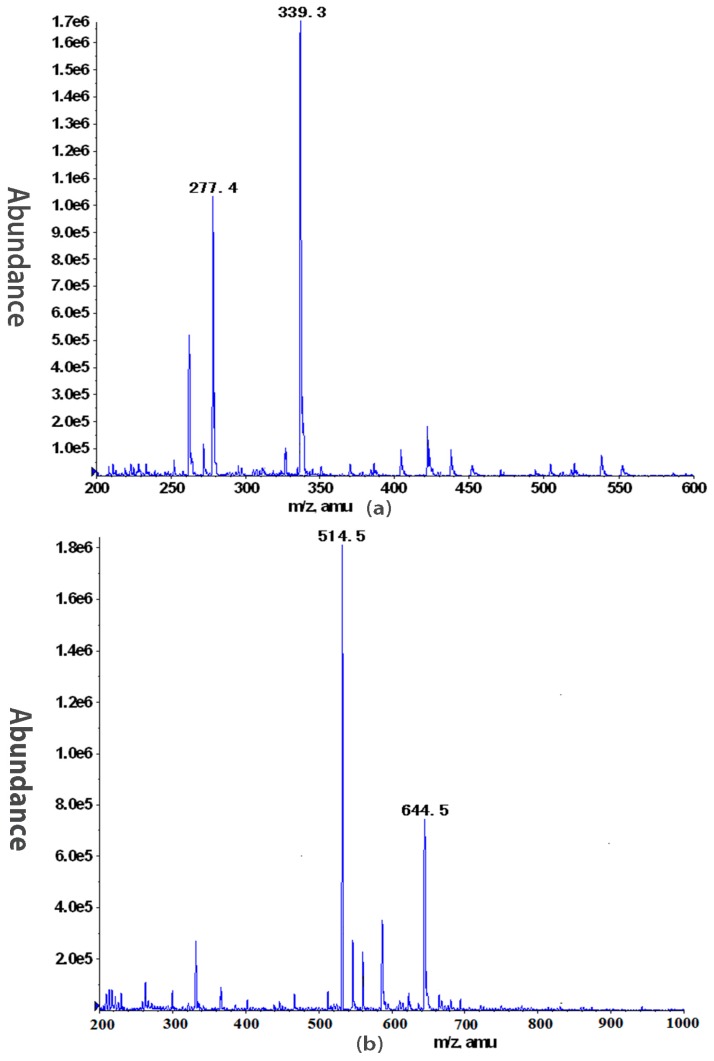
Negative ESI-MS of the mixture of disaccharide OB (**a**) and small oligosaccharide OC (**b**) obtained from DNP-W4.

Oligomers obtained by Smith degradation had been isolated on Sephadex G-10 to give OA, OB, OC and OD. OA was a mixture of monosaccharides. OB was a mixture of disaccharides. In ESI-MS (Figure 3a), ion fragments *m*/*z* 277.4 corresponded to [Glc − Glycerol + Na]^+^ and *m*/*z* 339.3 corresponded to [Rha − GalA − 1]^−^. OC was a mixture of tri- and tetra-saccharides, and in ESI-MS (Figure 3b), ion fragments *m*/*z* 514.5 and 644.5 corresponded to [GalA_2_ − Rha − 1]^−^ and [Gal_2_ − Rha − GalA − H_2_O]^−^ or [GalA_2_ − Rha_2_ − H_2_O − 1]^−^, respectively. OD was not investigated further. These results confirmed the existence of RG chains. And Xyl residues may be directly attached to *O*-2 of Rha.

Based on the results described above, it could be concluded that DNP-W4 possesses a backbone of (1→4)-linked β-d-Glc*p*, (1→6)-linked β-d-Glc*p*, and (1→6)-linked β-d-Gal*p*, with substitutes at *O*-4/6 of Glc*p* residues and *O*-3 of Gal*p*. The side chains may be composed of terminal Man*p*, (1→6)-linked β-d-Man*p*, (1→3)-linked β-d-Glc*p*, β-d-Glc*p*, (1→4)-linked α-d-GalA*p*, (1→2)-linked α-L-Rha*p*, and Xyl*p*.

### 2.3. Immunological Activity

The effects of the samples on the proliferation of ConA-and LPS-induced lymphocytes were tested in vitro (Table 3). In the immunological activity assay (Table 3), DNP-W4 had little activities even at the 100 μg/mL level, while DNP-W4E and HDNP-W4 had some proliferation enhancement at 50 μg/mL and 100 μg/mL dose on T and B lymphocytes. These results suggested that the side chains of DNP-W4 had effect on the expression of immunological activity. 

## 3. Experimental Section

### 3.1. Plant Materials and Chemical Reagents

The wild plant of *D. nobile* was purchased from Sichuan province of China in May 2007 and identified by Professor Luo Jian-ping of the School of Biotechnology and Food Engineering, Hefei University of Technology. A voucher specimen (No.: DNP0002) was stored in the herbarium of the School of Biotechnology and Food Engineering, Hefei University of Technology. DEAE–Cellulose, Sephacryl S-200, Sephadex G-10, pectolyase, NaBH_4_, concanavalin (ConA), lipopolysaccharide (LPS) and 3-(4,5-dimethylthiazol-2-yl)-2,5-diphenyltetrazolium brimoide (MTT) used were from Sigma-Aldrich (St. Louis, MO, USA). 1-Cyclohexyl-3-(2-morphlinoethy) carbodiimide metho-*p*-toluenesulphonate (CMC) and trifluoroacetic acid (TFA) were obtained from E. Merck (Darmstadt, Germany). T-series dextrans were acquired from Fluka (St. Gallen, Switzerland). All other reagents were available as analytical grade and were used without further purification. 

### 3.2. General Methods

The optical rotation and IR spectrum were recorded on a WZZ-1S polarimeter (Shanghai Physical Optics Instrument Co., Shanghai, China) and a Perkin Elmer 599B FT-IR spectrometer (Waltham, MA, USA), respectively. GC was done with a Shimadzu GC-9A instrument (Nishinokyo Kuwabaracho, Kyoto, Japan) equipped with a 3% OV-225 column (0.25 mm × 28 m i.d.) and FID. The column temperature for analysis was 250 °C. GC–MS was performed on a Trace GC2010/Trace MS chromatography (Nishinokyo Kuwabaracho) fitted with a fused silica capillary column of MXT-5 (0.25 μm × 0.25 mm × 30 m, Nishinokyo Kuwabaracho), and detected by FID (detector temperature 250 °C). d,l-configurations of glycosyl residues were obtained by the Gerwig method [29]. NMR spectra were carried out on a Bruker Avance DPX- 400 NMR spectrometer (Fällanden, Switzerland) with a dual probe in the FT mode. The Distortionless Enhancement by Polarization Transfer (DEPT) experiment was performed using a polarization-transfer pulse of 135°. The ESI-MS spectra were obtained with a API-3200 MS/MS spectrometer (Foster City, CA, USA) in reference to the literature [33]. 

### 3.3. Isolation and Purification

The fresh stems of *D. nobile* were dried in a drying cabinet at 50 °C The dried *D. nobile* was crushed into powder in a pulverizer before extraction. Pigments and fat were pervious removed from the powered materials with acetone and methanol; the remains were extracted with hot distilled water, deproteinated with a Sevag reagent [34], then extensively dialyzed with a dialysis bag in order to remove the hydronium and polysaccharide with small molecular weight (*M*_w_ cutoff 3500). The retentate was concentrated and precipitated with 4 vol of EtOH. The precipitate was dissolved with deionized water and then lyophilized to give the crude polysaccharide DNP-W. DNP-W (100 mg) was dissolved with deionized water and a portion of DNP-W was fractionated on a DEAE–Cellulose column (1.6 cm × 60 cm), and eluted stepwise with water, 0.05, 0.1, 0.2, 0.3, and 0.5 M NaCl. The subfraction obtained from 0.2 M NaCl elution was further purified on a Sephacryl S-200 column in order to obtain DNP-W4.

### 3.4. Gel-permeation Chromatography (GPC) and Molecular Mass

Measurements of the homogeneity and the molecular weight were performed by HPGPC with Waters 515 instrument, using a linked column of Ultrahydrogel™ linear column (7.8 mm × 300 mm, No. WAT011545) and Ultrahydrogel™ 500 column (7.8 mm × 300 mm, No. WAT11530, Waters, Milford, USA). The column was calibrated with standard T-series Dextrans (T-1000, T-500, T-100, T-50 and T-20). The specimen concentration was 1.5 mg/mL. The eluant was 0.005 M KNO_3_ and the flow rate was 0.5 mL/min.

### 3.5. Chemical Analysis

Neutral sugars of DNP-W4 were determined by GC after the hydrolyzate of DNP-W4 was transformed into alditol acetates, as described previously [16]. The uronic acid content was determined by the *m*-hydroxylbiphenyl method [28] and reduced before GC analysis. The reduction was carried out with the CMC–NaBH_4_ protocols [35], resulting in the carboxyl-reduced polysaccharide DNP-W4R. Methylation analysis was performed according to the method of Needs and Selvendran [36] after the methylated product was converted into partially methylated alditol acetates.

### 3.6. Partial Hydrolysis with Acid

DNP-W4 (300 mg) was hydrolyzed with 0.25 M TFA at 100 °C for 1 h. The mixture was cooled and dialyzed against distilled water. The retentate was lyophilized in order to obtain a degraded product HDNP-W4 and the dialysate was isolated using a Sephadex G-10 column; a main fraction (DP1) and a series of oligosaccharides were obtained.

### 3.7. Periodate Oxidation–Smith Degradation

15 mg DNP-W4 was oxidized using 0.015 M NaIO_4_ (20 mL) at 4 °C in the dark; the absorption was measured every day at 224 nm. The reaction was quenched with 2 mL glycol at the 7th day. Consumption of NaIO_4_ was measured by a spectrophotometric method [37] and HCOOH production was determined by titration with 0.005 M NaOH. The reaction mixture was reduced with NaBH_4_ (25 mg, 12 h), neutralized, dialyzed and lyophilized in order to obtain a degraded product DNP-W4-P. DNP-W4-P was further hydrolyzed with 0.2 M TFA at 40 °C for 24 h, and then dialyzed. The dialysate was further fractionated on a Sephadex G-10 column to give OA, OB, OC and OD, according to elution times.

### 3.8. Enzymatic Hydrolysis

DNP-W4 (15 mg) was dissolved with deionized water (3 mL), treated with a pectolyase solution at 37 °C for 2 h. After boiling in a dialysis bag at 100 °C for 15 min, the mixture was centrifuged and lyophilized in order to obtain DNP-W4E.

### 3.9. Immunobiological Activity Assay

Male BALB/c mice (18 ± 2 g, 8–10 weeks old) were obtained from the Experimental Animal Center, Anhui Medical University of China. After mice were sacrificed, their spleens were removed, minced, and passed through a sterilized ion mesh (200 mesh) to obtain single cell suspension, which was adjusted to a concentration of 5.0 × 10^6^ cells/mL with RPMI-1640 medium (Roswell Park Memorial Institute, USA). The cell suspension was seeded into a 96-well plate in the presence of the mitogen ConA (5.0 µg/mL) or LPS (10.0 µg/mL). Different dilutions of the polysaccharide samples (25 µg/mL, 50 µg/mL, 100 µg/mL) were incubated with the cell suspensions at 37 °C in a 5% CO_2_ atmosphere for 44 h, and then incubated for a further 6 h after the addition of 20 µL MTT. T-and B-lymphocyte proliferation was assayed by the MTT method [38]. The experiments were replicated three independent times.

### 3.10. Statistical Analysis

Dates were expressed as means ± standard deviation (SD). All statistical analyses were performed by using commercially available statistical software.

## 4. Conclusions

In this study, a water-soluble acidic heteropolysaccharide DNP-W4 was isolated from *Dendrobium nobile* Lindl. The molecular mass of DNP-W4 was 5.0 × 10^5^ Da in reference to standard dextrans. The sugar composition analysis revealed that DNP-W4 contained mannose, glucose, galactose, xylose, rhamnose, and galacturonic acid in the molar ratios of 1.0:4.9:2.5:0.5:1.0:0.9. The obtained data indicated that DNP-W4 was a complex heteropolysaccharide and possessed a backbone composed of (1→4)-linked β-d-Glc*p*, (1→6)-linked β-d-Glc*p*, and (1→6)-linked β-d-Gal*p*, with substitutes at *O*-4/6 of Glc*p* residues and *O*-3 of Gal*p*. The branches were composed of terminal Man*p*, (1→6)-linked β-d-Man*p*, (1→3)-linked β-d-Glc*p*, β-d-Glc*p*, β-d-Gal*p*, (1→4)-linked α-d-GalA*p*, (1→2)-linked α-L-Rha*p*, and Xyl*p*. The results of tests investigating immunological activity suggested that the side chains of DNP-W4 might have some positive effect on the expression of immunological activity.

## Figures and Tables

**Table 1:** Compositional analysis of DNP-W4 and its degraded polymers and oligomers (ratio).

Items	DNP-W4	DNP-W4E	HDNP-W4
Man	1.0	1.0	0.1
Glc	4.9	5.2	4.1
Gal	2.5	2.5	2.4
Xyl	0.5	n.d.	0.1
Rha	1.0	n.d.	0.1
GalA	0.9	n.d.	0.2

n.d., not detected.

**Table 2:** Linkage analysis of DNP-W4 and its derivatives (ratio).

Methylated Sugar	Linkages Types	DNP-W4	HDNP-W4	DP1
2,3,4,6-Me_4_-Glc*p*	β-d-Glc*p*-(1→	0.5	Trace	0.1
2,3,6-Me_3_-Glc*p*	→4)-β-d-Glc*p*-(1→	1.0	1.6	n.d.
2,3-Me_2_-Glc*p*	→4,6)-β-d-Glc*p*-(1→	1.0	0.1	n.d.
2,3,4-Me_3_-Glc*p*	→6)-β-d-Glc*p*-(1→	1.9	2.6	n.d.
2,4,6-Me_3_-Glc*p*	→3)-β-d-Glc*p*-(1→	0.5	n.d.	0.2
2,3,4-Me_3_-Gal*p*	→6)-β-d-Gal*p*-(1→	2.0	2.4	n.d.
2, 4-Me_3_-Gal*p*	→3,6)-β-d-Gal*p*-(1→	0.5	0.1	n.d.
2,3,4,6-Me_3_-Man*p*	β-d-Man*p*-(1→	0.5	0.1	0.2
2,3,6-Me_3_-Man*p*	→6)-β-d-Man*p*-(1→	0.6	Trace	0.2
2,3,4-Me_3_-Xyl*p*	Xyl*p*-(1→	0.5	0.1	0.4
3,4-Me_3_-Rha*p*	→2)-α-l-Rha*p*-(1→	1.0	0.1	1.0
2,3,6-Me_3_-GalA*p*	→4)-α-d-GalA*p*-(1→	0.9	0.2	1.0

n.d., not detected.

**Table 3:** Effects of DNP-W4 and its derivatives on proliferation of lymphocytes in vitro.

Items	DNP-W4 (OD_557_)		DNP-W4E (OD_557_)		HDNP-W4 (OD_557_)	
	ConA	LPS	ConA	LPS	ConA	LPS
Control	0.487 ± 0.06	0.540 ± 0.04	0.487 ± 0.06	0.540 ± 0.04	0.487 ± 0.06	0.540 ± 0.04
25 (μg/mL)	0.468 ± 0.01	0.527 ± 0.02	0.520 ± 0.05	0.553 ± 0.05	0.521 ± 0.07	0.559 ± 0.04
50 (μg/mL)	0.510 ± 0.05	0.577 ± 0.03	0.600 ± 0.02 *	0.600 ± 0.04	0.518 ± 0.04	0.581 ± 0.02
100 (μg/mL)	0.518 ± 0.08	0.573 ± 0.05	0.661 ± 0.05 **	0.686 ± 0.07 *	0.614 ± 0.03 *	0.635 ± 0.05 *

* *p* < 0.05, ** *p* < 0.01, significance from the control.
